# Genetic Insights Into Frailty: Association of 9p21-23 Locus With Frailty

**DOI:** 10.3389/fmed.2018.00105

**Published:** 2018-05-01

**Authors:** Sanish Sathyan, Nir Barzilai, Gil Atzmon, Sofiya Milman, Emmeline Ayers, Joe Verghese

**Affiliations:** ^1^Department of Neurology, Albert Einstein College of Medicine, Bronx, NY, United States; ^2^Department of Medicine, Albert Einstein College of Medicine, Bronx, NY, United States; ^3^Department of Genetics, Institute for Aging Research, Albert Einstein College of Medicine, Bronx, NY, United States; ^4^Department of Biology, Faculty of Natural Science, University of Haifa, Haifa, Israel

**Keywords:** frailty, fried index, 9p21-23 locus, aging, genetics

## Abstract

Frailty is a complex aging phenotype associated with increased vulnerability to disability and death. Understanding the biological antecedents of frailty may provide clues to healthy aging. The genome-wide association study hotspot, 9p21-23 region, is a risk locus for a number of age-related complex disorders associated with frailty. Hence, we conducted an association study to examine whether variations in 9p21-23 locus plays a role in the pathogenesis of frailty in 637 community-dwelling Ashkenazi Jewish adults aged 65 and older enrolled in the LonGenity study. The strongest association with frailty (adjusted for age and gender) was found with the SNP rs518054 (odds ratio: 1.635, 95% CI = 1.241–2.154; *p*-value: 4.81 × 10^−04^) intergenic and located between LOC105375977 and C9orf146. The prevalence of four SNPs (rs1324192, rs7019262, rs518054, and rs571221) risk alleles haplotype in this region was significantly higher (compared with other haplotypes) in frail older adults compared with non-frail older adults (29.7 vs. 20.8%, *p* = 0.0005, respectively). Functional analyses using *in silico* approaches placed rs518054 in the CTCF binding site as well as DNase hypersensitive region. Furthermore, rs518054 was found to be in an enhancer site of *NFIB* gene located downstream. *NFIB* is a transcription factor that promotes cell differentiation during development, has antiapoptotic effect, maintains stem cell populations in adult tissues, and also acts as epigenetic regulators. Our study found novel association of SNPs in the regulatory region in the 9p21-23 region with the frailty phenotype; signifying the importance of this locus in aging.

## Introduction

Frailty is a complex phenotype seen in aging, which is associated with low physiologic reserves and with increased vulnerability to adverse outcomes such as disability, hospitalization, and death (1, 2). The prevalence of frailty has been reported to range from 7 to 32% in older populations and is higher in women (3). Given the emergent aging pandemic worldwide (4), a major public health challenge is to find ways to enhance functional independence in older adults and to increase years free from disabilities. Hence, understanding the biological antecedents of frailty may provide insights into healthy aging strategies.

Frailty is a multidimensional construct involving several domains—physical, cognitive, psychological, and social domains (5–7). Even though expression and biomarker studies have pointed toward the involvement of various biological pathways in frailty (8, 9), genetic studies have not yielded consistent results. Candidate gene studies of *IL6, TNF* and *IGF1* have shown either no association with frailty or provided contradictory results (10). This might be mainly explained by the multifactorial nature of frailty with involvement of genetic, lifestyle, and epigenetic factors (11, 12). This multidimensionality and multifactorial or complex origin of frailty is further supported by the etiological overlap between frailty and various age-related complex or multifactorial disorders (13). Prevalent frailty was a strong risk factor for cardiovascular diseases (CVD) as well as associated mortality (14). In the Cardiovascular Health Study, a cross-sectional analyses showed 38% of frail individuals had prevalent heart disease compared with 17% in non-frail individuals (15). Frailty and diabetes are strongly linked (16) with a higher incidence of type 2 diabetes seen in individuals with frailty (17). Frailty is associated with postmortem Alzheimer pathology in older adults with and without an antemortem history of dementia (18, 19). All of these point toward overlapping biological mechanism for frailty and other complex disorders. It is also possible that complex disorders may alter the frailty risk conferred by specific biological pathways.

Complex or multifactorial diseases are caused by a combination of genetic, lifestyle, and other environment factors. Genome wide association studies (GWASs) have identified a large number of genetic variants associated with complex disorders (20, 21). In particular, 9p21-23 has been shown to be a risk-associated locus with many complex disorders. For example, *9p21* has been reported to be associated with CVD (22, 23), abdominal aortic aneurysm (24), arterial stiffness (25), peripheral artery disease (26), intracranial aneurysm (27), various types of cancers (28, 29), amyotrophic lateral sclerosis (30), primary open-angle glaucoma (29), vascular dementia, and Alzheimer’s disease (31). The *9p23* region was associated with restless legs syndrome (32) and obsessive-compulsive disorder (33). Distinct haplotype blocks at the 9p21-23 region were associated with CVD and type 1 diabetes (34). This locus harbors several genes including *ANRIL*, a long non-coding RNA gene implicated in the pathogenesis CVD and strokes, three candidate tumor suppressor genes; *CDKN2A* (cyclin-dependent kinase inhibitor 2A) encoding p16 protein, *CDKN2B* encoding p15 protein, and *p14/ARF* encoding p14ARF protein (35). *C9ORF72* gene was found to be associated with amyotrophic lateral sclerosis-frontotemporal dementia (36). Furthermore, protein tyrosine phosphatase receptor type delta (*PTPRD*) at 9p23 region was associated with restless legs syndrome (32) as well as cancers (37). While there is substantial overlap in the diseases-associated with frailty and the 9p21-23 locus, to the best of our knowledge, the association of this locus with frailty has not been specifically examined.

Discovering new biological pathways that prevent or delay frailty would increase current therapeutic options for clinicians and increase health span for individuals. Interestingly, rs2811712 located in *ANRIL* gene in the 9p21 locus is associated with physical function in older people with the minor allele being associated with reduced physical impairment (38). Furthermore, rs71321217 in *PTPRD* in the 9p23 locus is associated with gait rhythm (39). Based on these observations, we hypothesized that genetic variants in the chromosome 9p21-23 locus will increase the risk of developing frailty in older adults. To elucidate the role of the 9p21-23 locus in the pathogenesis of frailty, we conducted a preliminary cross-sectional study in 637 community-residing Ashkenazi Jewish (AJ) older adults participating in the LonGenity Study (40, 41). This population is homogenous genetically and socioeconomically (42) and allows for greater power for genetic analysis with fewer number of participants. Establishing the genetic underpinnings of frailty may provide new insights into preventive strategies to delay the occurrence of frailty and other related comorbidities as well as to promote healthy aging.

## Materials and Methods

### LonGenity Cohort

The LonGenity study, established in 2007, recruited a cohort of AJ adults age 65 and older, who were defined as either Offspring of Parents with Exceptional Longevity (OPEL) (having at least one parent who lived to age 95 or older) or Offspring of Parents with Usual Survival (OPUS) (neither parent survived to age 95). The goal of the LonGenity study is to identify genotypes associated with longevity and their association with successful aging. Study participants were recruited through contacts at synagogues, community organizations and advertisements in Jewish newspapers in the New York City area. Potential participants were contacted by telephone to assess interest and eligibility. They were invited to our research center for further evaluation. Exclusion criterion included diagnosis of dementia [previous physician diagnosed dementia or telephone Memory Impairment Screen scores in the dementia range (43)] as well as presence of severe visual or hearing impairments that would interfere with study assessments. Participants received detailed medical history evaluation and cognitive testing at baseline as well as at annual follow-up visits. All participants signed written informed consents for clinical assessments and genetic testing before enrollment. The Einstein institutional review board approved the study protocol.

A total of 965 older individuals were enrolled in the LonGenity study between October 2008 and August 2017. We excluded 64 individuals who did not complete frailty assessments as well as 264 who did not complete genetic testing. Hence, the eligible sample for this analysis included 637 participants, who had been genotyped and completed frailty assessments.

### Frailty Syndrome

The two common approaches to defining frailty clinically are as a clinical syndrome (5) or as a cumulative deficit score (44–46). The syndromic definition of frailty (see below) is widely adopted in research and clinical practice (5). While the cumulative deficit score approach has advantages in research settings, it is less intuitive in clinical settings in the community (47). Frailty diagnosis, hence, was operationalized using the widely used Cardiovascular Health Study criteria (48) for this study. Frailty was operationally defined as meeting three or more of the following five attributes: *unintentional weight loss* (≥10 lb in past year), *muscle weakness* (objectively measured grip strength or self-report; described below), *exhaustion* [negative response to the question “do you feel full of energy?” on the Geriatric Depression Scale (49)], self-reported *low physical activity* levels [positive response to the question “Have you been less active physically?” on the Health Self-Assessment Questionnaire (5)] and *slow gait* (Table 1). A Jamar handgrip dynamometer was used to objectively measure dominant hand grip strength at baseline. Weakness was defined using a cut score of 1 SD or more below age and sex mean values (Table 1). Similar to previous reports (50–52), subjective grip strength (“do you feel as though your grip is weak?”) was used on follow-up waves as a frailty criterion, since objective grip strength measures were not available for all our participants on follow-up. A previous study in this same cohort showed substantial agreement between the objective and subjective grip strength rating methods (53). Gait speed (cm/s) was measured using an 8.5 m long computerized walkway with embedded pressure sensors (GAITRite; CIR Systems, PA). The GAITRite system is widely used in clinical and research settings, and excellent reliability has been reported in our and other centers (54, 55). Participants were asked to walk on the walkway at their normal pace in a quiet well-lit room wearing comfortable footwear and without any attached monitors. Slow gait was defined as 1.5 or more SD below age and sex-appropriate mean values. In total, we had 206 individuals who were diagnosed with frailty; 118 prevalent cases and 88 incident cases of frailty.

**Table 1:** Clinical characteristics of cohort.

Variables	LonGenity	Frailty	Normal
Participants	637	206	431
Age, mean ± SD, years	75.41 ± 6.55	77.72 ± 6.75	74.29 ± 6.16
Women, %	52.9%	55.3	51.6
Education, mean, years	17.47 ± 2.70	17.33 ± 2.73	17.55 ± 2.68
Gait speed, mean ± SD, cm/s	110 ± 20.1	100.9 ± 21.4	114 ± 17.6
Offspring of Parents with Exceptional Longevity/Offspring of Parents with Usual Survival (%)	43.6/56.4	40.7/59.3	44.8/55.2
**Medical illnesses**			
Cardiovascular disease, %	9.1	12.30	7.50
Stroke, %	3.6	6.90	2.10
Diabetes, %	9.2	11.80	8.20
Parkinson disease, %	1.4	2.50	0.90
Arthritis, %	40.9	56.90	34.10
Hypertension, %	43.6	62.00	40.80
**Slow gait cuts, cm/s**			
Men <75 years	88.55		
Men ≥75 years	76.44		
Women <75 years	87.4		
Women ≥75 years	71.28		
**Low grip strength cuts, kg**			
Men <75 years	32.05		
Men ≥75 years	24.21		
Women <75 years	17.67		
Women ≥75 years	14.27		

### Selection of Gene Variants and Genotyping

We targeted 9p21-23 region spanning from chr9: 8743598 to 32586822 (NCBI build 37) for this analysis based on its functional significance and reported associations with major complex disorders (24, 30, 31, 34, 35). Genotyping was performed at the Center for Inherited Disease Research using Illumina HumanOmniExpress array (Illumina, San Diego, CA, USA), and the procedures have been described previously (40, 41).

Since the focus of our research was to explore complex disorder-associated alleles in this locus in regards to frailty; the few SNPs missing in the genotyping array were made available from imputation analysis. Imputation of un-genotyped autosomal SNPs were based on the 1000 Genomes data (worldwide reference panel of all 1,092 samples from the phase I integrated variant set) (v3, released March 2012) (56) using IMPUTE2, version 2.3.0. Poorly imputed SNPs with low imputation quality (info_metric < 0.3) were excluded from the analysis. For this study, we selected SNPs with minor allele frequencies of >0.10.

### Statistical Analysis

Baseline characteristics of participants were compared using descriptive statistics (Table 1). The preliminary objective of this study was to identify the association of variants in the 9p21-23 region with frailty using logistic regression analysis. Prevalent and incident cases of frailty were examined together in this analysis to maximize sample size. In participants who did not have frailty at baseline or develop incident frailty, the wave at which the first non-frail status was diagnosed was used as baseline for comparing clinical characteristics. As previous studies have shown frailty to increase with age and in women (57), all analyses were adjusted for age and gender (Model 1). All SNP based association analyses were conducted using Plink v1.90.^1^ All other statistical analyses were carried out using SPSS software (version 24; IBM Corporation). Presence or absence of diabetes, heart failure (including myocardial infarction, angina, or congestive heart failure), hypertension, strokes, Parkinson’s disease, and arthritis was used to calculate a global health score (range 0–6) as previously described (58). To account for the LonGenity study design described above (40, 41) and health status, we conducted sensitivity analyses further adjusting the models for OPUS/OPEL status and global health score (Model 2).

A total of 5,556 variants were available for analysis in the selected region (chr9: 8743598 to 32586822) after removing SNPs that had minor allele frequencies < 0.10 (*n* = 1,856) and failed the Hardy–Weinberg exact test (*p* ≥ 0.01) (*n* = 79). Linkage disequilibrium (LD) plots were generated using Haploview 4.2 (59). Haplotype blocks were defined based on the Gabriel criteria (60). Haplotype analyses were performed using SNPStats software (61). Functional prediction of the associated variants was carried out using various *in silico* approaches. Genotype-Tissue Expression portal (GTEx)^2^ was used to determine the significant expression quantitative trait loci (eQTL) for SNPs associated with frailty (62). Regulome DB^3^ based on Encyclopedia of DNA Elements (ENCODE) project (63) was used to identify functional effects of the identified SNPs in the association and eQTL analyses. rVarBase,^4^ updated database for regulatory features of variants was also used to find the effect of SNP of chromatin states, interacting regulatory elements and target genes (64). Functional Single Nucleotide Polymorphism; a web-based tool that integrates 16 databases and bioinformatic tools to uncover the functional effect of the SNPs (65) and FuncPred^5^ were used to predict the functional effects of associated variants.

## Results

### Study Population

Of the 637 eligible individuals with phenotype and genotype data in the LonGenity cohort, 356 were OPUS and 281 were OPEL. Of the eligible sample, 206 individuals (32.5%) received a diagnosis of frailty at baseline (*n* = 118) or at various time points over the study follow-up (*n* = 88), and 430 individuals (67.5%) remained non-frail throughout the study follow-up. The overall median follow-up time was 3.7 years (range 0–9 years). Demographic and clinical characteristics are summarized in Table 1. The mean age of the participants was 75.41 ± 6.55 years, and 52.9% were women. The mean years of education was 17.47 ± 2.70 years. A higher percentage of OPUS individuals met frailty diagnosis (34%) compared with OPEL (30.4%). All major medical illnesses were more prevalent in individuals who had frailty compared with normal individuals (Table 1).

### Association and *In Silico* Functional Analyses

**Figure 1:**
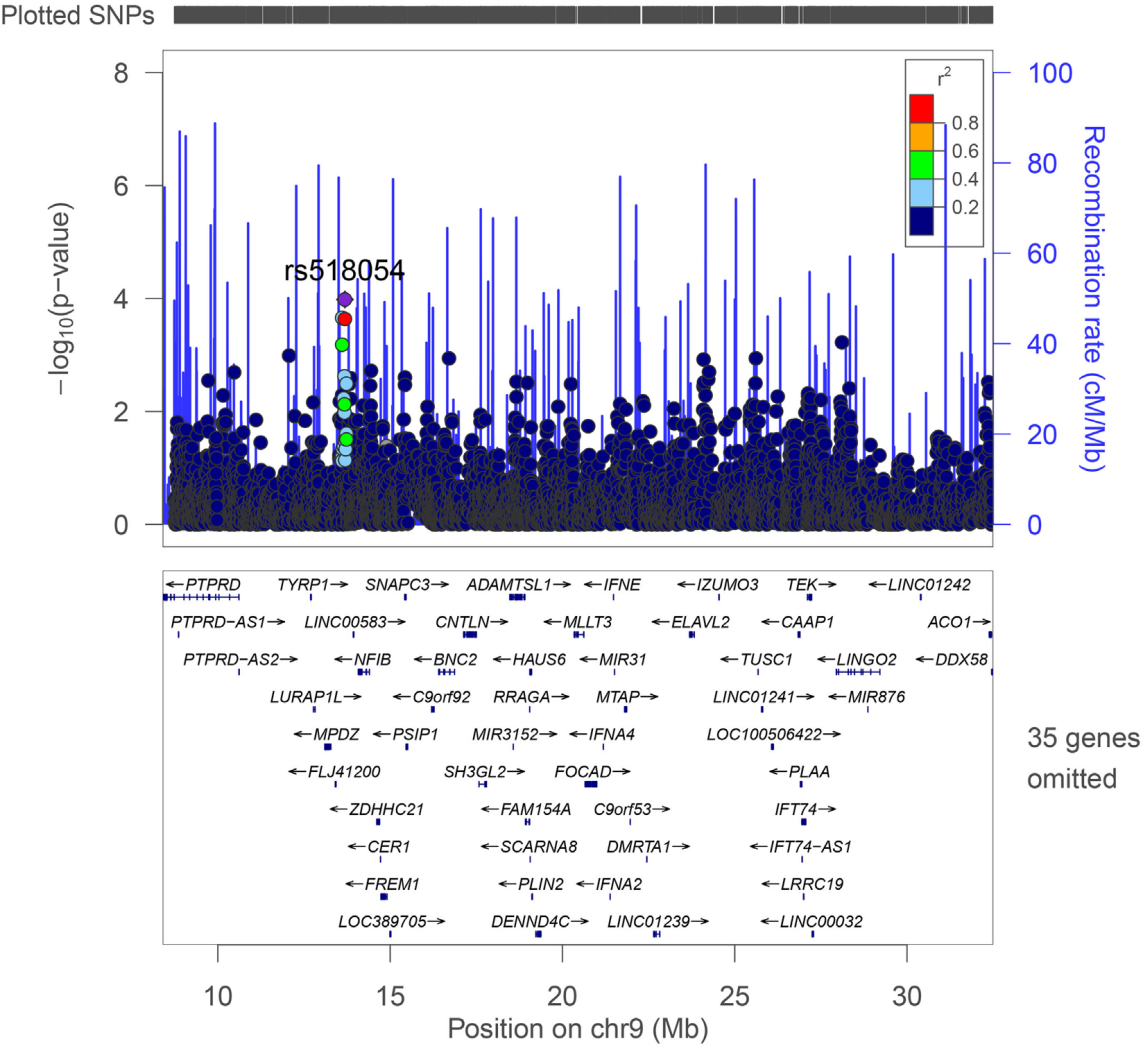
LocusZoom plot of the region studied with frailty on chromosome 9p21-23. Genes and ESTs within the region are shown in the lower panel, and the unbroken blue line indicates the recombination rate within the region. Each filled circle represents the *p*-value for one SNP, with the top SNP rs518054 shown in purple and SNPs in the region colored depending on their degree of correlation (*r*^2^) with rs518054 [as estimated internally by LocusZoom on the basis of CEU (Utah residents of Northern and Western European ancestry) HapMap haplotypes].

**Figure 2:**
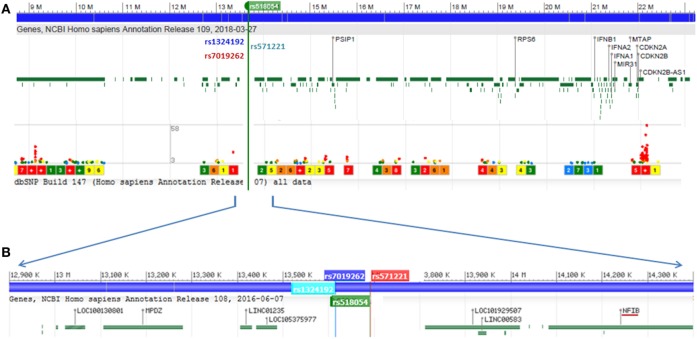
**(A)** Genome wide association study hotspot locus of 9p21-23 region screened in this study. Frailty-associated SNPs are marked in rsIDs, and lower dots indicate disease-associated SNPs in this region and level of significance. **(B)** Localized view of associated SNPs showing its location between LOC105375977 and LINC00583 (C9orf146). *NFIB* is the nearest well-characterized gene to SNP rs518054. Genomic region data adapted from NCBI dbSNP database.

The strongest association with frailty was found with the G allele of rs518054 [odds ratio (OR): 1.635, 95% CI = 1.241–2.154; *p*-value: 4.81 × 10^−04^] (Table 2; Figure 1) (Model 1). None of the SNPs studied survive Bonferroni correction for threshold for statistical significance. The associations remained similar after adjusting for longevity status (OPEL vs. OPUS) and global health score for all of these four SNPs (Table S1 in Supplementary Material) (Model 2). We also observed modest associations of three other SNPs (rs7019262, rs571221 and rs1324192) in this region with frailty (Table 2; Figure 2). LD plot of associated SNPs showed presence of SNPs in two LD blocks (rs518054–rs571221 and rs1324192–rs7019262) in frail and single LD block in normal individuals (Figure S1 in Supplementary Material). Haplotype analysis to investigate the combined effect of associated SNPs found significant association (*p*-value < 0.0005) with haplotype involving risk alleles (AGGC) at four loci combination (29.7 vs. 20.8%) (Table 3).

**Table 2:** Logistic regression analysis of 9p21-23 locus with Frailty with genotyped SNPs adjusted for age and gender (Model 1).

CHR	SNP	Position	Allele	Frail	Normal	STAT	Odds ratio (95% CI)	*p*
9	rs518054	13689066	G	0.314	0.214	3.491	1.635 (1.241–2.154)	4.81 × 10^−04^
9	rs10511667	18989696	G	0.164	0.106	3.411	1.855 (1.301–2.645)	6.48 × 10^−04^
9	rs1855850	10480030	T	0.329	0.419	−3.401	0.635 (0.489–0.825)	6.73 × 10^−04^
9	rs571221	13690235	C	0.314	0.219	3.341	1.597 (1.213–2.101)	8.35 × 10^−04^
9	rs7019262	13614384	G	0.510	0.400	3.330	1.517 (1.187–1.938)	8.68 × 10^−04^
9	rs7034231	28119512	G	0.186	0.115	3.254	1.780 (1.258–2.519)	1.14 × 10^−03^
9	rs1324192	13612345	A	0.483	0.383	3.176	1.488 (1.164–1.902)	1.50 × 10^−03^
9	rs7038172	16708269	C	0.147	0.087	3.125	1.802 (1.245–2.607)	1.78 × 10^−03^

*SNPs with p-value < 0.002 is shown in this table*.

**Table 3:** Haplotype analysis of the associated SNPs in the *9p21-23* region.

Sl. no.	rs1324192	rs7019262	rs518054	rs571221	Frailty	Normal	Odds ratio (95% CI)	*p*-Value
1	G	A	T	T	0.487	0.597	1	
**2**	**A**	**G**	**G**	**C**	**0.297**	**0.208**	**1.695 (1.266–2.273)**	**5 × 10^−04^**
3	A	G	T	T	0.184	0.173	1.300 (0.934–1.818)	0.12
4	G	G	T	T	0.012	0.011	1.010 (0.312–3.226)	0.99

*Significant difference in the haplotype (2) involving the risk alleles of associated SNPs was observed with 29.7% in individuals with frailty compared with 20.8% in normal*.

*Haplotype analysis was adjusted for age and gender*.

All four SNPs associated with frailty in this region were intergenic and located between LOC105375977 and C9orf146 (LINC00583) (Figure 2). The nearest well-characterized gene was *NFIB* coding for nuclear factor 1B (Figure 2). We assessed the functional significance of the associated SNPs in our study. The lead SNP rs518054 is located in the DNase 1 hypersensitive site. ENCODE data showed a *Regulome DB* score of 2b for rs518054, which predicted its role as likely to affect gene expression level, and the evidence includes transcription factor binding, any motif change, DNase footprint, and DNase peak (Table 4). The data show this region to be a binding site for CTCF, a transcriptional regulator. *rVarBase* data further suggest this SNP to be located in the chromatin interactive region with predominantly enhancer function in most tissues including muscle in both male and female. The available ENCODE data further showed this region harboring rs518054 interacted with the *NFIB* gene located downstream (Table 4). The associated SNP rs518054 located in the DNase hypersensitive site might play a role in the transcriptional regulation of *NFIB* gene through an enhancer effect. Furthermore, considering that these SNPs were located in the regulatory regions (e.g., enhancers), we used an *in silico* approach to determine whether they were local eQTL. Using GTEx portal, we could not find any significant eQTLs for SNP rs518054 in studied tissues.

**Table 4:** Details of putative regulatory functions of associated lead SNPs.

Variant	Ref	Alt	EUR freq	Promoter histone marks	Enhancer histone marks	DNAse	Proteins bound	Motifs changed	Chromatin state	Variant interacting gene	Frailty-associated cell line/tissue	Regulome Db score
**rs518054**	**T**	**G**	**0.20**	**–**	**10 tissues**	**10 tissues**	**CTCF**	**AIRE, Hoxb9**	**Enhancer**	**NFIB**	**Skeletal muscle**	**2b**
rs7019262	G	A	0.63	–	ESDR, LNG	MUS	P300	Pax-4, YY1	Enhancer	–	Skeletal muscle	4
rs571221	T	C	0.20	–	MUS	MUS, VAS	–	–	–	–	Skeletal muscle	5
rs10511667	A	G	0.89	–	Skin/lung	–	–	–	Enhancer	–	–	5
rs7034231	T	G	0.83	–	Neuron cells	–	–	–	Enhancer	–	–	5
rs7038172	T	C	0.94	–	Multiple tissues	–	GATA3 POLR2A	–	Enhancer	BNC2	–	6
rs1855850	C	T	0.67	–	–	–	–	–	–	–	–	–
rs1324192	A	G	0.66	–	–	–	–	–	–	–	–	–

*MUS, skeletal muscle, male; VAS, HUVEC umbilical vein endothelial primary cells; ESDR, H1 BMP4-derived trophoblast cultured cells; LNG, lung*.

*Data are derived from HaploReg v4.1 (http://www.broadinstitute.org/mammals/haploreg/haploreg.php), RegulomeDB (http://www.regulomedb.org/), and rVarBase (http://rv.psych.ac.cn/)*.

*Lead SNP rs518054 is marked in bold*.

Though none of the SNPs survived multiple corrections in this study, rs518054 emerged to be lead SNP with functional relevance in all models studied (Table 2; Table S1 in Supplementary Material). The unadjusted association analysis results are shown in Table S3 in Supplementary Material.

### Sensitivity Analyses

The next objective of our study was to find out the risk conferred by specific complex disorder-associated SNPs in this region with frailty. A number of CVD-associated SNPs were observed in the 9p21-23 locus followed by SNPs for cancers and many other complex disorders. Our analysis showed lack of association of these disease-associated SNPs with frailty (Table S2 in Supplementary Material). Interestingly, there was an increased prevalence of CVD-associated risk alleles [rs10757278 (*p* = 0.116), rs1333040 (*p* = 0.133), and rs1333049 (*p* = 0.116)] in frail individuals compared with non-frail individuals (Table S2 in Supplementary Material). SNPs associated with gait rhythm (rs71321217; *p*-value = 0.384) and physical activity (rs2811712; *p*-value = 0.205) in previous studies (38, 39) were not associated with frailty in our cohort (Table S2 in Supplementary Material).

Even though genetic studies have been carried out combining prevalent and incident cases (66–68), to check the possibility of survival bias arising from the possible systematic differences in allele frequencies between the prevalent and incident cases, we carried out case only analysis comparing allele frequency in incident and prevalent cases. There was slight difference in the allele frequency of rs518054 in incident and prevalent cases of frailty (*p*-value = 0.014) with associated G allele found to be 0.36 in prevalent cases and 0.25 in incident cases. The frequency of G allele in controls was 0.21. The overall association was mainly driven by cases of prevalent frailty (OR: 1.980, 95% CI = 1.426–2.749; *p*-value: 4.50 × 10^−05^) than incident frailty (OR: 1.198, 95% CI = 0.808–1.776; *p*-value: 0.368) when each of them were compared independently to controls adjusting for age and gender.

## Discussion

This study attempted to delineate the role of the 9p21-23 region with frailty in a well-characterized AJ cohort as a strategy to understand healthy aging. We uncovered a novel association of SNPs at the 9p21-23 region with frailty, not implicated previously with any of the complex disorders associated with this locus. Using functional analyses, we found the lead variant to be located in the enhancer region and involved in the transcriptional regulation of the *NFIB* gene. The study further observed increased frequency of CVD-associated alleles in individuals with frailty though failed to reach statistical significance with frailty phenotype.

The 9p21-23 region has emerged as a genetic hotspot for complex disorder associations in recent studies. With regard to the frailty-associated SNPs discovered in our study, the nearest well-characterized gene was *NFIB*, coding for the transcription factor Nuclear Factor IB, which plays a key role in the transcriptional regulation of a large number of genes in which our lead SNP rs518054 was found to be located in the enhancer region of this gene. *NFIB* has various functions ranging from promoting cell differentiation during development to maintaining stem cell populations in adult tissues and also possess antiapoptotic effect (69–72). *In vivo* studies have shown a multi-potency restriction of adult hippocampal neuronal stem cells by Drosha–NFIB interactions (73). It plays an important role in lung maturation and brain development (74), mediates repression of the epigenetic factor ezh2 which regulates cortical development (75), and also has an important role in chromatin remodeling (76). *NFIB* alters and globally maintains hyper accessible chromatin state and an increase of chromatin accessibility at distal regulatory elements enacts a program of gene expression (76). Thus the association we observed in the enhancer region of *NFIB* gene seems clinically and functionally relevant. The wide spread binding of *NFIB* in open chromatin sites has been linked to its regulatory action in adipocyte differentiation (77) and cancer metastasis (76). *NFIB* is also associated with osteosarcoma (78) and sciatica (79) in GWAS. All these findings point toward possible tissue-specific as well as genome-wide effects mediated through *NFIB*.

There is a paucity of studies examining the role of epigenetic mechanisms in frailty (12, 80). Epigenetic mechanisms including chromatin remodeling plays a pivotal role in the aging process (81, 82). Genes in the 9p21-23 locus have an important role in chromatin remodeling (76, 83). For instance, non-coding RNA *ANRIL*, specifically binds two polycomb proteins: CBX7 (PRC1) and SUZ12 (PRC2) to regulate histone modification in the CDKN2A/B locus. Overexpression of this gene also causes the down regulation of several genes involved in important chromatin architecture and remodeling mechanisms in other chromosomal regions (83). These results point toward a possible role of this locus in mediating environmental factors influenced epigenetic mechanisms. This might explain why this locus is linked with various age and environmental risk-associated diseases such as CVD, strokes and diabetes (24, 27, 34). Our finding thus supports a possible role of epigenetic mechanisms in frailty pathogenesis. Though there was higher prevalence of CVDs-associated risk allele with frailty, the association was not statistically significant. This might be mainly due to the smaller sample size as well as multifactorial origin of these diseases and frailty. Larger studies need to validate the initial observations in this study. Furthermore, since dementia was an exclusion criterion for the cohort, the association of some dementia related risk alleles with frailty might have minimized.

The strengths of our study include the systematic clinical and frailty assessments as well as the well-characterized population (40, 41). Limitation of this study is inclusion of incident frailty for increasing statistical power. The allele frequency of associated rs518054 “G” allele was observed to be more in prevalent and incident cases of frailty when independently compared with individuals free from frailty during course of this study. But the association was mainly driven by the prevalent frailty. The inclusion of incident frailty in the model provides us healthy controls free from frailty throughout the course of study. The lack of significant association with incident frailty might be mainly due to smaller sample size as well as objective-subjective definition of frailty used in this study. Limitations also include the absence of functional studies to validate the effect of associated genotypes with gene expression and chromatin interaction as well as the relatively small sample size. We noted the lack of consensus regarding frailty definitions, and chose a widely used and clinically relevant syndromic definition of frailty. Further studies need to be carried out to find the relevance of these observations in case of other frailty definitions. Our findings were based in a relatively genetically homogenous AJ population with high levels of education, which was used successfully for other genetic discoveries (40–42, 84–86) that have then been cross validated in other heterogeneous cohorts. However, our findings need to be validated in other more diverse populations.

In conclusion, we found novel association of variants in the 9p21-23 locus with frailty with lead SNP located in the enhancer region of the *NFIB* gene. Further investigation of this region is required to gain insights into potential interventions to address biological derangements in these pathways to extend health span and to maintain functional independence in older adults. The dynamics of healthy aging are complex and maintaining functional ability in older age is multifactorial. Frailty is one of the most significant geriatric syndrome observed in elderly population. Studies have shown that complex disorders increases with age but whether aging is the cause or consequence of these diseases is controversial. Our study supports a role for the complex disorder GWAS-associated 9p21-23 locus in frailty and provides insights into healthy aging.

## Ethics Statement

The study was approved by the Institutional Review Board at the Albert Einstein College of Medicine. Written informed consent was obtained from all the study participants in accordance with the Declaration of Helsinki.

## Author Contributions

SS and JV contributed to the design of the study and interpretation of the data. SS, NB, SM, and EA contributed to the acquisition of data and writing of the manuscript. EA, SM, and GA contributed to the analysis of the data. SS, NB, GA, SM, EA, and JV contributed to the critical revisions of the manuscript. All the authors approved the final version of the manuscript and agree to be accountable for all aspects of the work.

## Conflict of Interest Statement

The authors declare that the research was conducted in the absence of any commercial or financial relationships that could be construed as a potential conflict of interest.

## Footnotes

^1^https://www.cog-genomics.org/plink2.

^2^http://www.gtexportal.org/home/.

^3^http://regulomedb.org/.

^4^http://rv.psych.ac.cn/.

^5^https://snpinfo.niehs.nih.gov/snpinfo/snpfunc.html.
