# Supplementary material for: Genetic Insights Into Frailty: Association of 9p21-23 Locus With Frailty
Source: Front Med (Lausanne). 2018 May 1;5:105. doi: 10.3389/fmed.2018.00105 (PMC5938407; doi:10.3389/fmed.2018.00105)
Supplement: Supplementary file 1 [file Data_Sheet_1.docx]

Supplementary Material

**Genetic insights into healthy aging: Association of 9p21-23 locus with frailty**

Sanish Sathyan, PhD;^1^ Nir Barzilai, MD;^2,4^ Gil Atzmon, PhD;^2,3,4^ Sofiya Milman, MD;^2^ Emmeline Ayers, MPH;^1^ and Joe Verghese, MBBS, MS^1, 2, *^

**^1^** Department of Neurology, Albert Einstein College of Medicine, Bronx, New York, USA

**^2^** Department of Medicine, Albert Einstein College of Medicine, Bronx, New York, USA

^3^ Department of Biology, Faculty of Natural Science, University of Haifa, Haifa, Israel

^4^ Department of Genetics and the Institute for Aging research, Albert Einstein College of Medicine, Bronx, New York, USA

*** Correspondence:**

[Joe Verghese: joe.verghese@einstein.yu.edu](mailto:joe.verghese@einstein.yu.edu)

# Supplementary Figures and Tables

**Supplementary Table 1:** Logistic regression analysis of 9p21-23 locus with Frailty with genotyped SNPs adjusted for age, OPEL-OPUS status, sex and global health score (Model. 2).

| **CHR** | **SNP** | **Position** | **Allele** | **STAT** | **OR (95% CI)** | **P** |
| --- | --- | --- | --- | --- | --- | --- |
| 9 | **rs518054** | 13689066 | G | 3.663 | 1.701(1.280-2.259) | 2.49×10^-04^ |
| 9 | **rs571221** | 13690235 | C | 3.485 | 1.651(1.245-2.189) | 4.91×10^-04^ |
| 9 | rs10511667 | 18989696 | G | 3.354 | 1.867(1.296-2.689) | 7.96×10^-04^ |
| 9 | **rs7019262** | 13614384 | G | 3.270 | 1.523(1.184-1.960) | 1.08×10^-03^ |
| 9 | rs1359741 | 22336954 | G | -3.219 | 0.650(0.501-0.845) | 1.28×10^-03^ |
| 9 | rs10813796 | 32359091 | G | 3.186 | 1.516(1.174-1.957) | 1.44×10^-03^ |
| 9 | rs17221355 | 13857272 | T | -3.172 | 0.603(0.441-0.824) | 1.51×10^-03^ |
| 9 | rs1033991 | 26659562 | C | 3.157 | 1.574(1.188-2.086) | 1.60×10^-03^ |
| 9 | rs7042267 | 27135962 | T | 3.139 | 1.547(1.178-2.031) | 1.70×10^-03^ |
| 9 | rs10121064 | 26658407 | C | 3.137 | 1.567(1.184-2.074) | 1.71×10^-03^ |
| 9 | rs1023087 | 32376618 | G | -3.109 | 0.631(0.472-0.844) | 1.88×10^-03^ |
| 9 | rs986974 | 13815721 | C | -3.101 | 0.624(0.463-0.841) | 1.93×10^-03^ |
| 9 | rs1855850 | 10480030 | T | -3.100 | 0.653(0.499-0.855) | 1.93×10^-03^ |
| 9 | **rs1324192** | 13612345 | A | 3.087 | 1.487(1.156-1.912) | 2.01×10^-03^ |

SNPs with p-value <0.002 is shown in the table

**Supplementary Table 2:** Comparison of significant complex disorder associated SNPs in regard with frailty. The table shows the details of significant complex disorders associated SNPs in 9p21-23 locus and risk allele frequency in frail and healthy individuals in this study.

| **SNP** | **Position** | **Associated disease** | **Risk allele*** | **frail** | **Non-frail** | **OR; 95%CI; frailty** | **Frailty (p-value)** | |
| --- | --- | --- | --- | --- | --- | --- | --- | --- |
| rs1333040 | 22083404 | Myocardial infarction(Helgadottir et al., 2007), Intracranial Aneurysm(IA)(Bilguvar et al., 2008) | T | 0.715 | 0.673 | 1.217 (0.942-1.574) | | 0.133 |
| rs1333049^+^ | 22125503 | Coronary artery disease(Samani et al., 2007) | C | 0.645 | 0.599 | 1.217 (0.954-1.552) | | 0.114 |
| rs10757278^+^ | 22124477 | Myocardial infarction (Helgadottir et al., 2007), IA(Bilguvar et al., 2008) and Abdominal Aortic Aneurysm(AAA)(Helgadottir et al., 2008) | G | 0.640 | 0.594 | 1.215 (0.953-1.548) | | 0.116 |
| rs7023329 | 21816528 | Melanoma(Bishop et al., 2009) | A | 0.548 | 0.497 | 1.231 (0.973-1.557) | | 0.083 |
| rs564398 | 22029547 | Type 2 diabetes(Silander et al., 2009) | T | 0.739 | 0.704 | 1.194 (0.917-1.554) | | 0.187 |
| rs1412829^+^ | 22043926 | Glioma(Wrensch et al., 2009) | G | 0.261 | 0.297 | 0.837 (0.643-1.090) | | 0.187 |
| rs2383206 | 22115026 | Coronary heart disease(McPherson et al., 2007) | G | 0.691 | 0.661 | 1.149 (0.893-1.478) | | 0.280 |
| rs2383207 | 22115959 | Coronary Heart Disease(Helgadottir et al., 2007), AAA(Helgadottir et al., 2008) | G | 0.698 | 0.670 | 1.140 0.885-1.469) | | 0.311 |
| rs4626664 | 9261737 | Restless leg syndrome(Schormair et al., 2008) | A | 0.135 | 0.120 | 1.150 (0.811-1.630) | | 0.433 |
| rs2151280^+^ | 22034719 | Basal cell carcinoma(Stacey et al., 2009) | G | 0.379 | 0.395 | 0.934 (0.734-1.189) | | 0.581 |
| rs10811661^+^ | 22134094 | Type 2 diabetes(Silander et al., 2009) | T | 0.865 | 0.843 | 1.190 (0.850-1.666) | | 0.309 |
| **Frailty component associated SNPs** | | | | | | | | |
| rs71321217^+^ | 10036568 | Gait rhythm(Adams et al., 2015) | Ins T | 0.193 | 0.173 | 1.143 (0.846-1.545) | | 0.384 |
| rs2811712 | 21998035 | Physical function(Melzer et al., 2007) | A | 0.882 | 0.905 | 0.785 (0.540-1.142) | | 0.205 |

**Supplementary Table 3:** Association analysis of 9p21-23 locus with Frailty with genotyped SNPs (unadjusted)

| **CHR** | **SNP** | **Position** | **Ref. allele** | **Frail** | **Normal** | **Alt.** | **OR (95% CI)** | **P** |
| --- | --- | --- | --- | --- | --- | --- | --- | --- |
| 9 | rs518054 | 13689066 | G | 0.314 | 0.214 | T | 1.682(1.292-2.190) | 1.04×10^-04^ |
| 9 | rs7019262 | 13614384 | G | 0.510 | 0.400 | A | 1.559(1.231-1.974) | 2.18×10^-04^ |
| 9 | rs571221 | 13690235 | C | 0.314 | 0.219 | T | 1.636(1.258-2.129) | 2.29×10^-04^ |
| 9 | rs7034231 | 28119512 | G | 0.186 | 0.115 | T | 1.756(1.270-2.429) | 5.95×10^-04^ |
| 9 | rs1324192 | 13612345 | A | 0.483 | 0.383 | G | 1.508(1.190-1.911) | 6.48×10^-04^ |
| 9 | rs12686721 | 12067552 | C | 0.188 | 0.120 | A | 1.706(1.238-2.352) | 1.01×10^-03^ |
| 9 | rs7038172 | 16708269 | C | 0.147 | 0.087 | T | 1.809(1.261-2.594) | 1.13×10^-03^ |
| 9 | rs10511761 | 25612704 | T | 0.500 | 0.404 | G | 1.478(1.168-1.872) | 1.14×10^-03^ |
| 9 | rs4416909 | 24095294 | C | 0.428 | 0.524 | T | 0.677(0.535-0.858) | 1.20×10^-03^ |
| 9 | rs10810148 | 14447766 | G | 0.362 | 0.277 | A | 1.485(1.157-1.907) | 1.88×10^-03^ |
| 9 | rs17771619 | 24262091 | T | 0.167 | 0.243 | C | 0.623(0.461-0.842) | 2.00×10^-03^ |

SNPs with p-value <0.002 is shown in the table

**Supplementary Figure 1:** LD plot of associated genotyped SNPs in individuals with frailty and normal

**
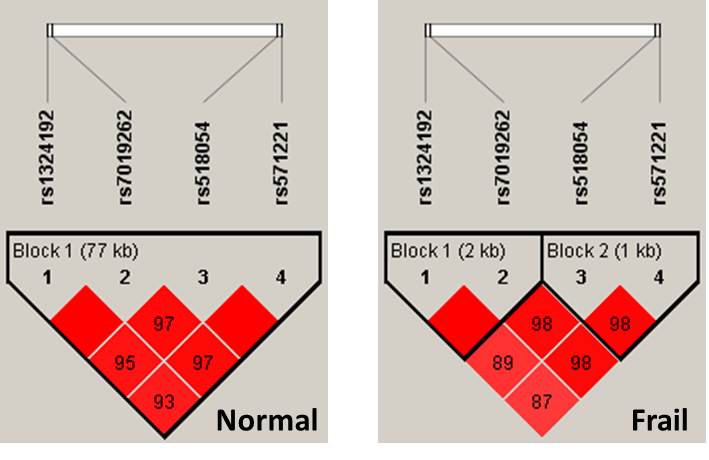
**

Adams, H.H., Verlinden, V.J., Callisaya, M.L., van Duijn, C.M., Hofman, A., Thomson, R., et al. (2015). Heritability and genome-wide association analyses of human gait suggest contribution of common variants. *The Journals of Gerontology Series A: Biological Sciences and Medical Sciences***,** glv081.

Bilguvar, K., Yasuno, K., Niemelä, M., Ruigrok, Y.M., von und zu Fraunberg, M., van Duijn, C.M., et al. (2008). Susceptibility loci for intracranial aneurysm in European and Japanese populations. *Nature genetics* 40(12)**,** 1472-1477.

Bishop, D.T., Demenais, F., Iles, M.M., Harland, M., Taylor, J.C., Corda, E., et al. (2009). Genome-wide association study identifies three loci associated with melanoma risk. *Nature genetics* 41(8)**,** 920-925.

Helgadottir, A., Thorleifsson, G., Magnusson, K.P., Grétarsdottir, S., Steinthorsdottir, V., Manolescu, A., et al. (2008). The same sequence variant on 9p21 associates with myocardial infarction, abdominal aortic aneurysm and intracranial aneurysm. *Nature genetics* 40(2)**,** 217-224.

Helgadottir, A., Thorleifsson, G., Manolescu, A., Gretarsdottir, S., Blondal, T., Jonasdottir, A., et al. (2007). A common variant on chromosome 9p21 affects the risk of myocardial infarction. *Science* 316(5830)**,** 1491-1493.

McPherson, R., Pertsemlidis, A., Kavaslar, N., Stewart, A., Roberts, R., Cox, D.R., et al. (2007). A common allele on chromosome 9 associated with coronary heart disease. *Science* 316(5830)**,** 1488-1491.

Melzer, D., Frayling, T.M., Murray, A., Hurst, A.J., Harries, L.W., Song, H., et al. (2007). A common variant of the p16 INK4a genetic region is associated with physical function in older people. *Mechanisms of ageing and development* 128(5)**,** 370-377.

Samani, N.J., Erdmann, J., Hall, A.S., Hengstenberg, C., Mangino, M., Mayer, B., et al. (2007). Genomewide association analysis of coronary artery disease. *New England Journal of Medicine* 357(5)**,** 443-453.

Schormair, B., Kemlink, D., Roeske, D., Eckstein, G., Xiong, L., Lichtner, P., et al. (2008). PTPRD (protein tyrosine phosphatase receptor type delta) is associated with restless legs syndrome. *Nature genetics* 40(8)**,** 946-948.

Silander, K., Tang, H., Myles, S., Jakkula, E., Timpson, N.J., Cavalli-Sforza, L., et al. (2009). Worldwide patterns of haplotype diversity at 9p21. 3, a locus associated with type 2 diabetes and coronary heart disease. *Genome medicine* 1(5)**,** 51.

Stacey, S.N., Sulem, P., Masson, G., Gudjonsson, S.A., Thorleifsson, G., Jakobsdottir, M., et al. (2009). New common variants affecting susceptibility to basal cell carcinoma. *Nature genetics* 41(8)**,** 909-914.

Wrensch, M., Jenkins, R.B., Chang, J.S., Yeh, R.-F., Xiao, Y., Decker, P.A., et al. (2009). Variants in the CDKN2B and RTEL1 regions are associated with high-grade glioma susceptibility. *Nature genetics* 41(8)**,** 905-908.
